# State Endowment of Unqualified Practice

**Published:** 1914-02-07

**Authors:** 


### State Endowment of Unqualified Practice.

THE concluding paragraphs of the above report deal with a matter which is of immense importance to the State, its insured citizens, and the medical profession. This extraordinary regulation not merely recognises but actually proposes to endow unqualified practice in this country. It has already aroused a storm of protest, for it is one of the most vicious developments of an Act which has already done far too much in introducing ill-considered and ill-advised changes in our social system. The National Medical Union has done well to act promptly, and it is to be hoped that the General Medical Council will take the hint which the Union has offered.

When it is remembered that thousands of the insured have been refused permission to make their own arrangements for medical attendance with qualified practitioners; and that this refusal of rights plainly contemplated in the Act as indisputably theirs has been purely and simply a political dodge, designed to force medical men on to the panels, this coquetting with quackery is intelligible only on the supposition that some party advantage is expected from it. What this can be is none too plain, but, unfortunately, it is clear as daylight that the insured will suffer.

If anyone wants proof of this, certain legal proceedings now pending at Bedford are convincing enough. As reported in the local press, a man named Delvine has been charged with obtaining money under false pretences in a village near Bedford. The man has been remanded on this charge, and has also been arrested on some other charge of a different nature. It would therefore be improper for us to express any opinion on the evidence as at present published as to his guilt or innocence; and for our present purpose it is quite unnecessary to do so. It is sufficient to say that he is not a qualified medical man, that he claims to be a cancer-curer, and that the charge of fraud is in connection with this claim.

Now in the village where he has been practising this man Delvine has gained the confidence of many of the peasantry, who bitterly resent his arrest and the proceedings against him. Many of them believe that he has cured them of cancer, and the local practitioners are hooted and molested when out of doors. One of them even has to receive police protection. All this because the people think that the doctors are actuated by jealousy of the "cancer-curer." Whether this ill-feeling will survive the result of the trial remains to be seen. The important points for the moment are that unqualified practice is apparently to be directly remunerated and recognised by the State; that unqualified practitioners are free of the restraints which bind the qualified; that the more unblushing the effrontery and the more blatant the advertisements of the unqualified the larger their clientèle is likely to be; and that large sections of the insured classes are totally unable to distinguish truth from falsehood in medical affairs.

### PANEL WORK AND DOCTORS' INCOMES.

During the last few days several interesting tables of figures have been published in various parts of the country, showing the general run of the incomes that are being made by panel practitioners. The figures are somewhat surprising in some respects, and considerable differences are shown between various towns. Broadly speaking, they do not support the view, now so energetically pressed in the Ministerialist party press, that the medical profession as a body (or rather the panel sections of it) is making a huge fortune out of the Act.

In Birmingham, for instance, it has been shown that out of 150 panel practitioners, 110 earn less than £175 a year out of panel work: so says the *Manchester Guardian*, which candidly admits also that in return for increased pay the doctors in working-class districts are doing a great deal more work than they ever did before. Twelve of the panellers in Birmingham earn upwards of £1,050 out of Insurance work; and this means, of course, that those practitioners have accepted more names than they can possibly attend to properly.

In Leeds, again, the average panel income is calculated at £300 a year; but this figure includes some men with very few patients, so that the average earnings of those who go in for panel practice seriously may be rather higher. In Glasgow, out of 380 panel doctors, 150 earn on an average £70 a year each; eighty-seven more earn on an average £280 a year each; and there are but thirteen whose gains amount to £845 or upwards. Manchester has similar figures to show, though medical attendance on the insured is worked there on a different plan from that which prevails elsewhere. At this latter city an attempt is being made to enforce a limit of £800 a year for each individual practitioner; but, as was stated last week in these columns, it is doubtful whether this is possible.

